# Dissipative MHD free convective nanofluid flow past a vertical cone under radiative chemical reaction with mass flux

**DOI:** 10.1038/s41598-023-28702-0

**Published:** 2023-02-18

**Authors:** E. Ragulkumar, G. Palani, P. Sambath, Ali J. Chamkha

**Affiliations:** 1 Department of Mathematics, SRM Institute of Science and Technology, Kattankulathur,Chennai, Tamil Nadu 603203 India; 2 Department of Mathematics, Dr. Ambedkar Govt. Arts College, Chennai, Tamil Nadu 600039 India; 3 Faculty of Engineering, Kuwait College of Science and Technology, 35004 Doha District, Kuwait

**Keywords:** Applied mathematics, Scientific data, Chemical engineering, Mechanical engineering, Mathematics and computing

## Abstract

Recently, Nanoparticles have supplied diverse challenges to several scientific issues. Nanoparticles dispersed in a variety of conventional fluids can change the flow and heat transmission properties of the fluids. The mathematical technique is used in this work to investigate the MHD water-based nanofluid flow via an upright cone. The heat and mass flux pattern is used in this mathematical model to examine MHD, viscous dissipation, radiation, chemical reactions and suction/injection processes. The finite difference approach was used to find the solution to the basic governing equations. A combination of nanofluids comprising nanoparticles including aluminum oxide (Al$_{2}$O$_{3}$), silver (Ag), copper (Cu) and titanium dioxide (TiO$_{2}$) with a volume fraction of nanoparticles (0, 0.01, 0.02, 0.03, 0.04), viscous dissipation ($\epsilon = 0.4, 0.8$), MHD (M = 0.5, 1.0), radiation (Rd = 0.4, 1.0, 2.0), chemical reaction ($\lambda = 0.2, 2.0$) and heat source/sink ($\Delta = -3, -2 ,0.5 , 1$) . The mathematical findings of velocity, temperature, concentration, skin friction, heat transfer rate as well as Sherwood number distributions are analyzed diagrammatically using non-dimensional flow parameters. It has been discovered that by increasing the value of the radiation parameter, velocity and temperature profiles enhance. The production of safe, high-quality products for consumers across the world depends on vertical cone mixers, from food to medicine, household cleansers to personal hygiene products. Every vertical cone mixer type we provide was especially developed to meet the demands of industry. As the mixer warms up on the slanted surface of the cone while vertical cone mixers are being utilized, the effectiveness of the grinding may be felt. The temperature is transferred along the cone’s slant surface as a consequence of the mixture being mixed quickly and repeatedly. This study describes the heat transmission in these events and their parametric properties. The heated cone’s temperature is then convective to its surroundings.

## Introduction

The term “nano” was originally used in 1915 by Oswald^1^ on his book “The World of Neglected Dimensions.” Nanotechnology is a trendy research subject in the 21st century due to the unique property of matter at the nano-scale. Made in recent decades, researchers as well as scientists from all over the world have attempted to research on numerous aspects of nanotechnology on a constant basis. The suspension of metallic and non-metallic particles in conventional fluids can significantly improve heat transfer performance. The development of nanotechnology and related manufacturing techniques has allowed for the production of nanosized particles. Nanofluids are fluids containing nanomaterials (width just under 100 nm) in conventional heat transfer fluids, as defined by Choi S.U.S.^2^ to improve heat transport properties. The ultimate focus of nanofluids is to attain the highest achievable effect on thermal conductivity while using the fewest nanoparticles possible. A nanofluid gained advantages such as the ability to conduct heat more efficiently, to cool microchannels without clogging and to pump more efficiently due to its enhanced thermal conductivity. Gupta et al.^3^ investigated a Cattaneo-Christov analysis of the heat and mass fluxes affecting the MHD Jeffrey liquid after it passes through a permeable cone. The stability analysis was used by Annur et al.^4^ to explore the effect of buoyancy force on the permeability moving plate along in carbon nanotubes. A study undertaken by Sambath et al.^5^ addressed the ruling PDE’s for the transient radiative MHD heat and mass transfer flow past an upright cone when a chemical reaction is taking place and derived numerical solutions based on Crank-Nicholson methods. Hanifa Hanif et al.^6^ studied variable viscosities in water-based hybrid nanofluid flows in a cone with an inverted permeable cone while heat generation/absorption. In order to perform the numerical analysis, we have to take the existent magnetic field and radiative heat flux into account. The theoretical effect of Brownian motion on the natural convection flow of nanoparticles along a circular cone were studied by Iqbal et al.^7^. The work of Kannan et al.^8^ discussed laminar convective fluid flow with a vertical cone with an electrically conducting fluid flow generated by surface heat flux and magnetic field. Hanif et al.^9^ investigated the two-dimensional flow of a water-based nanofluid including non-spherical CdTe nanoparticles solution across an inverted cone. Thameem Basha et al.^10^ investigated the chemical reaction of nanofluids in two different geometries based on electrohydromagnetic and nonuniform heat sources/sinks. Saleem et al.^11^ studied the flow of a Walter’s B nanofluid on the revolving cone in the presence of magnetic fields. The angular velocity near and far from the cone should be a reverse linear curve of time. An effect on electromagnetohydrodynamics has indeed been investigated by Vijayalakshmi et al.^12^ for a chemically reacting Casson fluid with two different configurations. The impacts of Lorentz force were studied by HT Basha et al.^13^ on a chemically reacting nanofluid with two different configurations to understand the properties of fluid transport. Abdul gaffar et al.^14^ focused on the influence of radiative MHD on a third-grade viscoelastic non-Newtonian fluid exterior to an isothermal vertical cone. The behavior of heat and mass transfer is investigated by Sulochana et al.^15^ for magneto-hydrodynamic streams across a vertical rotating cone with thermal radiation and chemical processes. Researchers PS Reddy et al.^16^ used a vertical cone filled with a porous nanofluid to investigate heat as well as mass transport properties. Sreedevi et al.^17^ surveyed both heat and mass transfer study of water-based nanoparticles comprising single and multi-walled CNTs along a vertical cone immersed in a porous medium exhibiting convective boundary conditions under the impact of a chemical process as well as suction/injection. HT Basha et al.^18^ studied two different types of configurations to describe hydro-magnetic nanofluid flow involving higher-order chemical reactions as well as non-uniform heat source/sink. A viscous thermally accelerating nanofluid flow was investigated by R Vemula et al.^19^ by applying a vertical plate with a changing temperature as well as thermal radiation that is subjected to the magnetic field The heat exchanger mechanism was established by S Nandal and R Bhargava^20^ in a two-dimensional stable natural convection flow of a nanofluid around an inclined plate. As a result, the nonlinear convection as well as radiation, were presented by Mahanthesh et al.^21^ on tangent hyperbolic fluid flow across a convectively heated vertical surface. PS Reddy et al.^22^ investigated the heat as well as mass transmission properties of an magnetic field nanofluid along with a slanted vertical plate submerged into a permeable substance containing heat radiation as well as a heat generating component. Abdul Gaffar et al.^23^ studied the MHD plain convective flow, heat and mass transfer of viscoelastic immiscible Jeffrey’s fluid across a vertical cone including the effects of heat radiation and heat generation/absorption. A water based aluminum alloy nanofluid with inclined magnetic field and electrical conductivity was explored by Sandeep and Animasaun^24^. Using a vertical cone and a flat plate saturated with non-Darcy porous medium, Durairaj et al.^25^ have analyzed Casson flow generating or absorbing heat by chemical reaction. Specifically, Sridevi et al.^26^ have critiqued the convective boundary condition with suction/injection for MHD boundary layer heat transfer. PS Reddy et al.^27^ explored the effect of thermal radiation as well as chemical reaction on the transmission of thermal as well as mass in a plain convective nanofluid peripheral layer flow along a up straight cone. A vertical uniform magnetic field and thermal radiation interact analytically in an experiment by M Turkyilmazoglu et al.^28^ to influence free convection of nanofluids flowing over a horizontal infinite isothermal plate. PS Reddy and AJ Chamkha^29^ illustrated the plain convective Peripheral layer heat as well as mass transport properties of nanofluids around a vertical cone using two types as well as sizes of nanoparticles. N Sandeep and MG Reddy^30^ illustrated the mathematical model by considering nonlinear thermal radiation as well as heat source/sink influence to examine the heat transport design nature of electrically conducting MHD nanofluid flow over a cone and a wedge. CSK Raju et al.^31^ studied the role of Brownian motion and thermophoresis in nanofluids in the presence of non-uniform heat suction/injection effects as well varying MHD fields over a cone. B Mallikarjuna et al.^32^ examined integrated thermal as well as mass transmission mostly in presence of a magnetic field including chemical reaction effects using mixed convection flow of a Newtonian fluid through a revolving vertical cone immersed in a porous media. IS Oyelakin et al.^33^ analyzed the Cattaneo-Christov Casson nanofluid with variable viscosity flow over a vertical cone in the effect of Brownian motion immersed in the porous medium.

The unsteady Casson fluid flow over a vertical cone and a flat plate was studied by Jasmine Benazir et al.^34^ and the influence of double dispersion, non-uniform heat source and sink as well as higher order chemical reactions. Several types of nano-particles, base fluids and working temperatures were considered by Abolfazl Zaraki et al.^35^ to determine how natural convection heat and mass transfer occur over flat plates. The boundary layer flow of such a MHD Eyring-Powell nanofluid through a permeability cone has been examined by Jayachandra Babu et al.^36^ in terms of buoyancy effects and suction/injection effects. A study by Raju et al.^37^ investigated how thermophoresis and Brownian motion impact the boundary layer flow in a MHD Jeffrey nanofluid over a permeable cone. A solution of the natural convective MHD with mass transfer flow over a vertical cone has been obtained by Sambath et al.^38^ and its governing equation has been derived by the Crank-Nicolson method. Sambath et al.^39^ investigated the flow of a viscous, electrically conductive, incompressible fluid over a vertically inclined plate while both a heat source and sink existed. The heat transfer features of natural convection were examined by Palani and Kim^40^ utilizing a vertical cone when thermal radiation and a magnetic field were present. Ganesan and Palani^41^ conducted a detailed computational investigation of MHD flow across a semi-infinite inclined plate with changing surface heat as well as mass flux. Uniform surface heat flux was first proposed in the literature by Lin^42^ to determine the flow of laminar plain convection from an upright circular cone with constant wall flux. A plumb cone with uneven surface heat flux was examined by Hossain and Paul^43^ to determine how laminar plain convective peripheral layers current. The effects of Arrhenius activation energy and binary reactions on heat and mass transfer in magnetohydrodynamic Jeffrey fluid flow on the surface of the stretching sheet are explored^44^ in the presence of non-uniform heat generation, thermal radiation, and velocity slip. A chemical reaction over an autocatalytic stretching sheet is investigated by Samuel^45^ with regard to the effects of temperature-dependent viscosity on radiant Maxwell fluid flow. It is the first attempt at free convection nanofluid flow across a vertical cone surface with various parameters with heat as well as mass flux. A vertical cone substance is submerged in a nanoparticle-containing fluid and examined how the heat and mass flux in the nanofluid takes place rather than in the fluid where the heat as well as mass transfer from the cone occurs.

The following section discusses whether heat and mass transfer occurrence changes with the addition of fluid parameters. Moreover, we have shown graphically how thermal and mass flow conditions impact nanofluid properties during heat and mass transfer.

## Formulation of the problem

### Problem description

**Figure 1:**
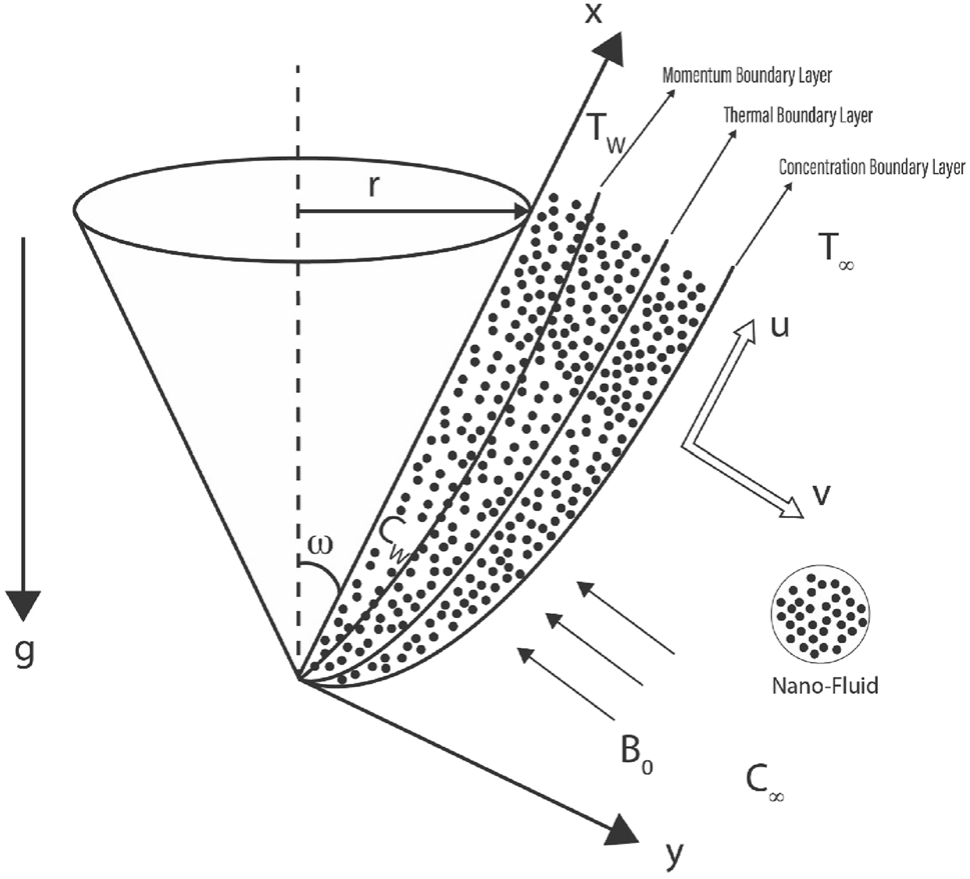
Physical model and coordinate system.

The magnetohydrodynamic radiative nanofluid flow across a up straight cone has been explored using a non-uniform heat source/sink and MHD in the present work. The influence of viscous dissipation, chemical reaction and thermal radiation has been included in the current study to control the heat and mass flux. The radius and half-angle of the cone are determined by r and $\omega$, respectively, and the flow develops in an upward direction as a result. The x-axis is drawn parallel to the surface of the cone and the y-axis is drawn normal to it, as shown in the flow configuration diagram in Fig. 1. Assuming that the ambient temperature and concentration are always below Tw and Cw, then Tw > $T_{\infty }$ and Cw > $C_{\infty }$. The constant ambient temperature as well as concentration far away from the surface, are represented by the numerals $T_{\infty }$ and $C_{ \infty }$. The combination of thermal and species buoyancy terms is the first and second upon the right side of velocity Eq. (2), whereas the hydromagnetic drag term is the last component. The second component on the right side of temperature Eq. (3) is associated with thermal radiation, the third term is the heat source/sink term and last term is viscous dissipation term. The final term in diffusion Eq. (4) correlates to a first order chemical process.

### Governing equation (flow analysis)

In accordance with Researchers^5,40^ approach, the equations for continuity, momentum, energy and spices can be expressed as follows:

Equation of Continuity

1 $$\begin{aligned} \dfrac{\partial (ru)}{\partial x}+\dfrac{\partial (rv)}{\partial y}=0 \end{aligned}$$

Equation of Momentum

2 $$\begin{aligned} \dfrac{\partial u }{\partial t'}+ u \dfrac{\partial u }{\partial x}+ v \dfrac{\partial u }{\partial y} = g \dfrac{(\rho \beta _{T})_{nf}}{\rho _{nf}} \big (T'-T'_{\infty }\big )cos\omega + g \dfrac{(\rho \beta ^{*}_{C})_{nf}}{\rho _{nf}} \big (C'-C'_{\infty }\big )cos\omega + \nu _{nf}\dfrac{\partial ^{2} u}{{\partial y}^{2}}-\dfrac{\sigma B^{2}_{0}}{\rho _{nf}}u \end{aligned}$$

Equation of Energy

3 $$\begin{aligned} \dfrac{\partial T'}{\partial t'}+ u \dfrac{\partial T' }{\partial x}+ v \dfrac{\partial T'}{\partial y} = \dfrac{1}{(\varrho c_{p})_{nf}}\bigg [k_{nf} \dfrac{\partial ^{2}T'}{{\partial y}^{2}} + \mu _{nf} {\bigg (\dfrac{\partial u}{\partial y}\bigg )^{2}} -\dfrac{\partial q _{r}}{\partial y} +Q_{0}(T'-T'_{\infty }) \bigg ] \end{aligned}$$

Equation of Concentration

4 $$\begin{aligned} \dfrac{\partial C'}{\partial t'}+ u \dfrac{\partial C'}{\partial x}+ v \dfrac{\partial C'}{\partial y} = D \dfrac{\partial ^{2} C'}{{\partial y}^{2}}-K_{1}(C'-C'_{\infty }) \end{aligned}$$

Initial and boundary condition are

5 $$\begin{aligned}{} & {} t'\le 0: u=0, v=0, T'=T'_{\infty }, C'=C'_{\infty }\text {~~~for~all~~ x~~ and ~~y }\nonumber \\{} & {} \quad t'>0: u=0, v=0, ~~\dfrac{\partial T'}{\partial y}=\dfrac{-q_{w}x}{k},\dfrac{\partial C'}{\partial y}=\dfrac{-q^{*}_{w}x}{D}~~~~~at~~y=0\nonumber \\{} & {} \quad u=0, ~~T'=T'_{\infty }~~~~~~C'=C'_{\infty }~~~~~~~~~~~~~~~at~~x=0\nonumber \\{} & {} \quad u\rightarrow 0, ~~T'\rightarrow T'_{\infty }~~~~~C'\rightarrow C'_{\infty }~~~~~~~~~~~~~~~~~as~~y\rightarrow \infty \end{aligned}$$

where $$q_{w}(x)= ax^{n}, q^{*}_{w}(x)= bx^{n}$$

Here a and b is the constants, $B^{2}_{0}$ is the magnetic field induction, C$_{p}$ is the specific heat at constant pressure, C$'$ is the concentration in the fluid, C$'_{\infty }$ is the concentration far-away from cone surface, D is the thermal diffusivity, g is the acceleration due to gravity, k is the thermal conductivity, L is the reference length, T$^{'}$ is the temperature, T$'_{\infty }$ is the temperature far-away from cone surface, t$^{'}$ is the time, u and v are the velocity components along the x and y directions (dimensional), respectively, $\beta$ is the volumetric coefficient of thermal expansion, $\mu$ is the dynamic viscosity, $\mu _{nf}$ is the dynamic viscosity of the nanofluid, $\nu$ is the kinematic viscosity, $\nu _{f}$ is the base fluid of the kinematic viscosity, $\sigma$ is electrical conductivity, $\omega$ is the cone apex half-angle.

**Table 1:** Thermo-physical properties^19,46^ of water and nanoparticles.

Physical features	Water	Copper	Silver	Aluminum oxide	Titanium dioxide
$\bar{\rho } (kg/m^{3})$	997.10	8933.0	10500	3970.0	4250.0
$\bar{C}_{\bar{p}} (J/kgK)$	4179.0	385.0	235	765.0	686.2
$\bar{k} (W/mk)$	0.613	401.0	429	40.0	8.9538
$\bar{\beta } \times 10^{-5} (K^{-1})$	21.0	1.67	1.89	0.85	0.90

The density $\rho _{nf}$, thermal expansion co-efficient $(\rho \beta )_{nf}$, nanofluid of the thermal conductivity ($k_{nf}$) and heat capacitance $(\rho c_{p})_{nf}$ a expressions for nanofluids are presented.

$$\begin{aligned}{} & {} \rho _{nf}=(1-\phi )\rho _{f}+\phi \rho _{s}\\{} & {} \quad (\rho \beta )_{nf}=(1-\phi )(\rho \beta )_{f}+\phi (\rho \beta )_{s}\\{} & {} \quad (\rho c_{p})_{nf}=(1-\phi )(\rho c_{p})_{f}+\phi (\rho c_{p})_{s}\\{} & {} \quad \dfrac{k_{nf}}{k_{f}} =\dfrac{k_{s}+(n-1)k_{f}-(n-1)\phi (k_{f}-k_{s})}{k_{s}+(n-1) k_{f}+ \phi (k_{f}-k_{s})}\\{} & {} \dfrac{\mu _{nf}}{\mu _{f}}=^{} \dfrac{1}{(1-\phi )^{2.5}} \end{aligned}$$

The relevant non-dimensional quantities are introduced as follows:

$$\begin{aligned}{} & {} X=\dfrac{x}{L},~~ Y=\dfrac{y}{L} Gr^{1/5}_{L},~~ U = \dfrac{uL}{\nu _{f}} Gr^{-2/5}_{L},~~ V = \dfrac{vL}{\nu _{f}} Gr^{-1/5}_{L}\\{} & {} \quad t=\dfrac{\nu _{f} t' (Gr_{L})^{2/5}}{L^{2}},~~T=\dfrac{T'-T'_{\infty }}{(q_{w}(L)L/k)}Gr_{L}^{1/5} ,~~~C=\dfrac{C'-C'_{\infty }}{(q_{w}(L)L/D)}Gr_{L}^{1/5},\\{} & {} \quad Gr_{L}= \dfrac{(g\beta _{T})_{f} q_{w}(L)L^{4}cos\omega }{k_{f}~\nu ^{2}_{f}}, Gr_{C}= \dfrac{(g\beta _{c}^{*})_{f} q_{w}^{*}(L)L^{4}cos\omega }{\nu ^{2}_{f} ~ D_{f}}, \lambda =\dfrac{K_{1}L^{2}}{\nu _{f}} Gr_{L}^{-2/5}\\{} & {} \quad Pr=\dfrac{\nu _{f}}{\alpha _{f}},M = \dfrac{\sigma B^{2}_{0}L^{2}}{\mu _{f}} Gr_{L}^{-2/5}, Sc=\dfrac{\nu _{f}}{D}, \Delta =\dfrac{Q_{0}L^{2}}{\nu _{f}(\rho c_{p})_{f}}Gr_{L}^{-2/5}\\{} & {} \quad R_{d} = \dfrac{k_{1}^{*} k}{4 \sigma ^{*} T^{'3}_{\infty }}, N=\dfrac{Gr_{C}}{Gr_{L}}, \epsilon =\dfrac{g\beta L}{(c_{p})_{f}},~~R =\dfrac{r}{L};~~~r= x~ sin~\omega ; \end{aligned}$$

where, Gr$_{L}$ is the dimensionless thermal Grashof number, Gr$_{C}$ is the dimensionless mass Grashof number, Pr is the Prandtl number, N is dimensionless ratio due to buoyancy force, $Q_{0}$ is the dimensional heat source/sink, R is dimensionless local radius, r is the local radius of the cone, Sc is the Schmidt number, U and V are the velocity components along the X and Y directions (dimensionless), respectively, T is the dimensionless temperature, t is the dimensionless time.

We use the Rosseland approximation^38^ to model unidirectional radiative heat flux, which yields the following formula regarding radiative heat flux qr:

6 $$\begin{aligned} q_{r}= - \dfrac{4 \sigma ^{*}}{3K^{*}}\dfrac{\partial T ^{4}}{\partial y} \end{aligned}$$

The linear version of Eq. (7) may be obtained by rising T$^{'}$ $^{4}$ using the expansion of Taylor series over T$^{'}_{\infty }$ while deleting upper-order components if the term T$^{'}$- T$^{'}_{\infty }$ inside the flow is sufficiently small.

7 $$\begin{aligned} T^{4}_{\infty } \cong 4 T^{ 3}_{\infty } T-3T^{4}_{\infty } \end{aligned}$$

Substituting Eqs. (6) and (7) in Eq. (3), we have

8 $$\begin{aligned} \dfrac{\partial T'}{\partial t'}+ u \dfrac{\partial T' }{\partial x}+ v \dfrac{\partial T'}{\partial y} = \dfrac{1}{(\varrho c_{p})_{nf}}\bigg [k_{nf} \dfrac{\partial ^{2}T'}{{\partial y}^{2}} + \mu _{nf} {\bigg (\dfrac{\partial u}{\partial y}\bigg )^{2}} +Q_{0}(T'-T'_{\infty }) - \dfrac{16 \sigma ^{*}T^{3}_{\infty }}{3K^{*} }\dfrac{\partial ^{2} T}{\partial y^{2}} \bigg ] \end{aligned}$$

The governing equations are provided in the non-dimensional form below.

Equation of Continuity

9 $$\begin{aligned} \dfrac{\partial U}{\partial X} + \dfrac{\partial V}{\partial Y} + \dfrac{U}{X}= 0 \end{aligned}$$

Equation of Momentum

10 $$\begin{aligned} \dfrac{\partial U}{\partial t}+ U \dfrac{\partial U}{\partial X}+V\dfrac{\partial U}{\partial Y}= A_{1} \bigg [ A_{2} (T) + A_{3} \big (NC-MU\big ) \bigg ] + A_{4} \dfrac{\partial ^{2} U}{{\partial Y}^{2}} \end{aligned}$$

Equation of Energy

11 $$\begin{aligned} \dfrac{\partial T}{\partial t}+ U \dfrac{\partial T}{\partial X}+V\dfrac{\partial T}{\partial Y}= A_{5} \bigg [ \dfrac{k_{nf}}{k_{f}} \dfrac{1}{Pr}\dfrac{\partial ^{2} T}{{\partial Y}^{2}} - \dfrac{1}{Pr} \bigg (\dfrac{3R_{d}+4}{3R_{d}}\bigg ) T + \Delta T \bigg ] + A_{6} ~~\epsilon {\bigg (\dfrac{\partial U}{\partial Y}\bigg )^{2}} \end{aligned}$$

Equation of Concertration

12 $$\begin{aligned} \dfrac{\partial C}{\partial t}+ U \dfrac{\partial C}{\partial X}+V\dfrac{\partial C}{\partial Y}= \dfrac{1}{Sc}\dfrac{\partial ^{2} C}{{\partial Y}^{2}}-\lambda C \end{aligned}$$

where, $$A_{1} = \dfrac{1}{(1-\phi )+\phi \bigg (\dfrac{\rho _{s}}{\rho _{f}}\bigg )} ,A_{2} = \bigg ((1-\phi )+\phi \dfrac{(\rho \beta )_{s}}{(\rho \beta )_{f}}\bigg ), A_{3} = (1-\phi )+\phi \dfrac{(\rho \beta ^{*})_{s}}{(\rho \beta ^{*})_{f}}$$ , $$A_{4} = \dfrac{1}{(1-\phi )^{2.5}\bigg (1-\phi +\phi \dfrac{\rho _{s}}{\rho _{f}}\bigg )} , A_{5} = \dfrac{1}{(1-\phi )+\phi \dfrac{(\rho c_{p})_{s}}{(\rho c_{p})_{f}}}, A_{6} = \dfrac{1}{(1-\phi )^{2.5}\bigg (1-\phi +\phi \dfrac{(\rho c_{p})_{s}}{(\rho c _{p})_{f}}\bigg )}$$

Initial and boundary conditions in non dimensional form are

13 $$\begin{aligned}{} & {} t'\le 0: ~~U=0, V=0, T=0 \text { for all X and Y}\nonumber \\{} & {} \quad t'>0: ~~U=0, V=0, ~~\dfrac{\partial T }{\partial Y} = -X^{n},~~\dfrac{\partial C}{\partial Y}=-X^{m}~~~at~~Y=0\nonumber \\{} & {} \quad U=0, ~T=0,~C=0~~~~~~~~~~~~~~~~~~~~~~~~~~~at~~X=0\nonumber \\{} & {} \quad U\rightarrow 0 ~~T\rightarrow 0~~C\rightarrow 0~~~~~~~~~~~~~~~~~~~~~~~~~~~as~~Y\rightarrow \infty \end{aligned}$$

### Engineering curiosity

Mathematically, the non-dimensional co-efficient of local skin friction ($\tau _{X}$), the local Nusselt number ($Nu_{X}$) and the local Sherwood number $Sh_{X}$ are difined as

$$\begin{aligned}{} & {} {\tau _{X}}=\dfrac{1}{(1-\phi )^{2.5}} Gr^{3/5}_{L}\bigg (\dfrac{\partial U}{\partial Y}\bigg )_{Y=0}\\{} & {} \quad Nu_{X}=\dfrac{k_{nf}}{k_{f}} \dfrac{-X\bigg (\dfrac{\partial T}{\partial Y}\bigg )_{Y=0}Gr^{1/5}_{L}}{T_{Y=0}}\\{} & {} \quad Sh_{X}= \dfrac{-X\bigg (\dfrac{\partial C}{\partial Y}\bigg )_{Y=0}Gr^{1/5}_{L}}{C_{Y=0}} \end{aligned}$$

Also, the non-dimensional average skin friction ($\overline{\tau }$), the average Nusselt number ($\overline{N_{u}}$) and the average Sherwood number ($\overline{Sh}$) are difined as:

$$\begin{aligned}{} & {} \bar{\tau }=\dfrac{2}{(1-\phi )^{2.5}} Gr^{3/5}_{L}\int _{0}^{1}X\bigg (\dfrac{\partial U}{\partial Y}\bigg )_{Y=0}~~dX\\{} & {} \quad \overline{N_{u}}=2\dfrac{k_{nf}}{k_{f}}Gr^{1/5}_{L}\int _{0}^{1} \dfrac{-X\bigg (\dfrac{\partial T}{\partial Y}\bigg )_{Y=0}}{T_{Y=0}}~~dX\\{} & {} \quad \overline{Sh}=2 Gr^{1/5}_{L}\int _{0}^{1} \dfrac{-X\bigg (\dfrac{\partial C}{\partial Y}\bigg )_{Y=0}}{C_{Y=0}}~~dX \end{aligned}$$

## Computational solution

In this article, we use implicit finite difference schemes of the Crank-Nicolson type in order to solve PDEs (9) to (12) involving initial as well as boundary conditions (13) . This numerical technique starts by converting the above PDE’s (9) to (12) into finite difference equations by using the corresponding finite difference operator with grid discretization and these equations are given by:

14 $$\begin{aligned}{} & {} \dfrac{U^{k+1}_{i,j} - U^{k+1}_{i-1,j}+ U^{k}_{i,j}-U^{k}_{i-1,j} + U^{k+1}_{i,j-1}-U^{k+1}_{i-1,j-1}+U^{k}_{i,j-1}-U^{k}_{i-1,j-1}}{4 \Delta X} \nonumber \\{} & {} \quad +\dfrac{V^{k+1}_{i,j} -U^{k+1}_{i,j-1}+ U^{k}_{i,j}-U^{k}_{i,j-1}}{2 \Delta Y } + \dfrac{U^{k+1}_{i,j} -U^{k+1}_{i,j-1}+ U^{k}_{i,j} - U^{k}_{i,j-1}}{4 i \Delta X } = 0 \end{aligned}$$

15 $$\begin{aligned}{} & {} \dfrac{U^{k+1}_{i,j} -U^{k}_{i,j}}{\Delta t} + U^{k}_{i,j} \dfrac{U^{k+1}_{i,j}-U^{k+1}_{i-1,j} + U^{k}_{i,j}-U^{k}_{i-1,j}}{2 \Delta X} + V^{k}_{i,j} \dfrac{U^{k+1}_{i,j+1}-U^{k+1}_{i,j-1} + U^{k}_{i,j+1}-U^{k}_{i,j-1}}{4 \Delta Y} \nonumber \\{} & {} \quad = A_{1} \bigg [ \dfrac{ A_{2} }{2} \big (T^{k+1}_{i,j} +T^{k}_{i,j}\big ) + \dfrac{ A_{3} }{2} \bigg ( N \big (C^{k+1}_{i,j} +C^{k}_{i,j}\big ) - M \big (U^{k+1}_{i,j} +U^{k}_{i,j}\big )\bigg )\bigg ] \nonumber \\{} & {} \quad + A_{4} \dfrac{U^{k+1}_{i,j-1} - 2 U^{k+1}_{i,j}+ U^{k+1}_{i,j+1} + U^{k}_{i,j-1} - 2 U^{k}_{i,j} + U^{k}_{i,j+1}}{2 (\Delta Y) ^{2}} \end{aligned}$$

16 $$\begin{aligned}{} & {} \dfrac{T^{k+1}_{i,j}-T^{k}_{i,j}}{\Delta t}+U^{k}_{i,j} \dfrac{T^{k+1}_{i,j}-T^{k+1}_{i-1,j}+T^{k}_{i,j}-T^{k}_{i-1,j}}{2 \Delta X} + V^{k}_{i,j} \dfrac{T^{k+1}_{i,j+1}-T^{k+1}_{i,j-1}+T^{k}_{i,j+1}-T^{k}_{i,j-1}}{4 \Delta Y} \nonumber \\{} & {} \quad = A_{5} \bigg [ \dfrac{k_{nf}}{k_{f}} \dfrac{1}{Pr} \bigg (\dfrac{T^{k+1}_{i,j-1} - 2 T^{k+1}_{i,j}+ T^{k+1}_{i,j+1} + T^{k}_{i,j-1} - 2 T^{k}_{i,j} + T^{k}_{i,j+1}}{2 (\Delta Y) ^{2}}\bigg ) \nonumber \\{} & {} \quad - \dfrac{1}{Pr} \bigg (\dfrac{3R_{d}+4}{3R_{d}}\bigg ) \big (T^{k+1}_{i,j} +T^{k}_{i,j}\big ) + \dfrac{ \Delta }{2} \big (T^{k+1}_{i,j} +T^{k}_{i,j}\big ) \bigg ]\nonumber \\{} & {} \quad + A_{6} ~~\epsilon \bigg (\dfrac{U^{k+1}_{i,j+1}-U^{k+1}_{i,j-1} + U^{k}_{i,j+1}-U^{k}_{i,j-1}}{4 \Delta Y}\bigg )^{2} \end{aligned}$$

17 $$\begin{aligned}{} & {} \dfrac{C^{k+1}_{i,j} -C^{k}_{i,j}}{\Delta t} + U^{k}_{i,j} \dfrac{C^{k+1}_{i,j}-C^{k+1}_{i-1,j} + C^{k}_{i,j}-C^{k}_{i-1,j}}{2 \Delta X} + V^{k}_{i,j} \dfrac{C^{k+1}_{i,j+1}-C^{k+1}_{i,j-1} + C^{k}_{i,j+1}-C^{k}_{i,j-1}}{4 \Delta Y} \nonumber \\{} & {} \quad = \dfrac{1}{Sc} \dfrac{C^{k+1}_{i,j-1} - 2 C^{k+1}_{i,j}+ C^{k+1}_{i,j+1} + C^{k}_{i,j-1} - 2 C^{k}_{i,j} + C^{k}_{i,j+1}}{2 (\Delta Y) ^{2}}- \dfrac{ \lambda }{2} \big (C^{k+1}_{i,j} +C^{k}_{i,j}\big ) \end{aligned}$$

Where (i, j) indicates the grid location in the x and y directions, respectively. As assumed that, $\Delta X$, $\Delta Y$ and $\Delta t$ refer to the step sizes in X, Y and t, respectively, while (n, n + 1) refers to the n$^{th}$ and (n+1)$^{th}$ iterations stand for U, T and C, respectively. The finite difference equations of velocity, temperature and concentration are obtained by using properly chosen constant values. After getting finite difference equations, we must transform them into algebraic equations. The Thomas algorithm is used to solve these algebraic equations, which are provided in the form of a tridiagonal system. In this numerical technique, the step size in the $\Delta X$ and $\Delta Y$ directions is 0.05, with a time step of $\Delta t$ =0.01 while X$_{max}$ = 1 and Y$_{max}$ = 20 reflect the boundary conditions corresponding to $y\rightarrow \infty$. The steady-state condition attained when the measured variable satisfied within the tolerance limit $10^{-5}$.

## Result and discussion

**Figure 2:**
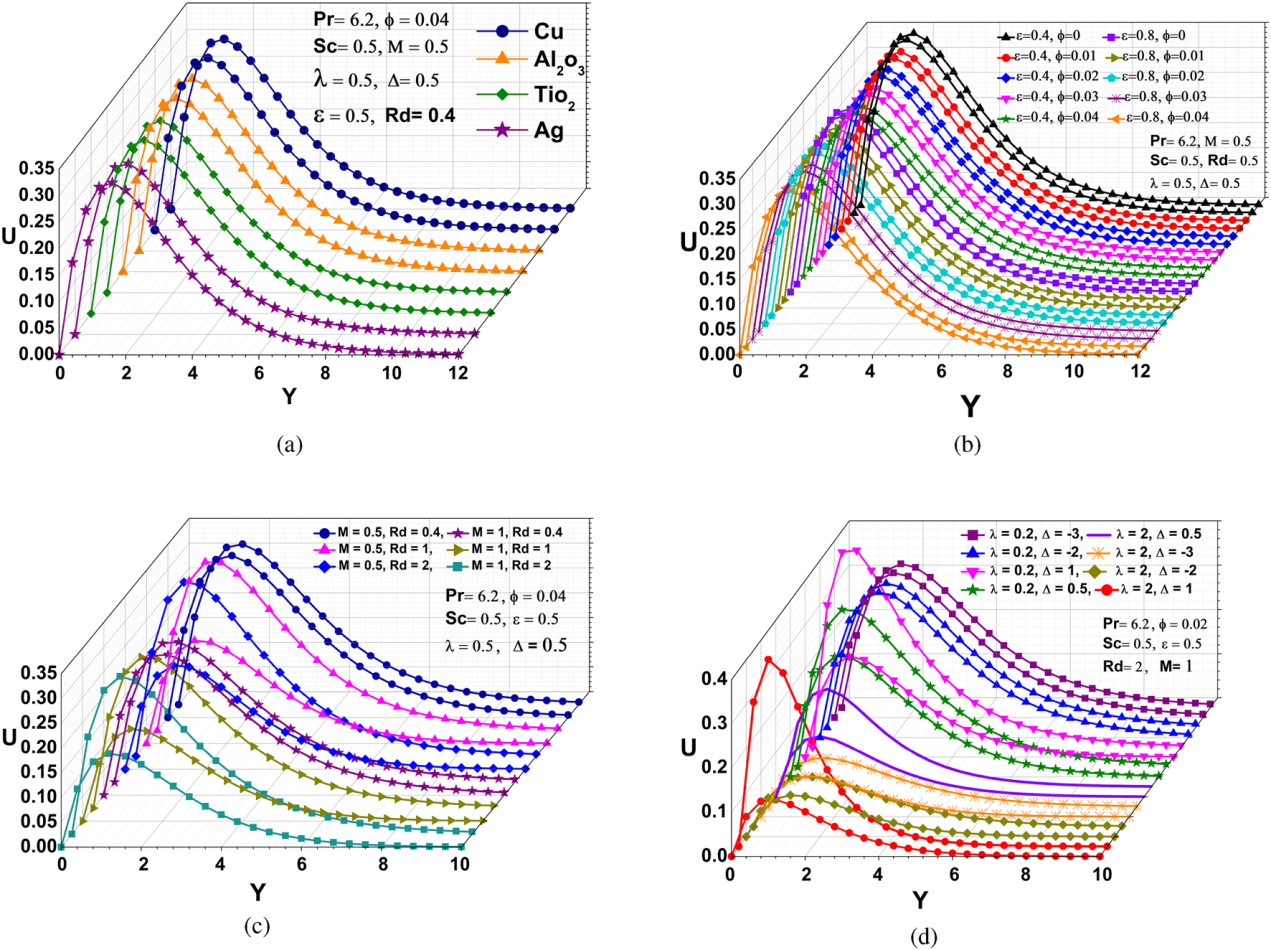
Velocity profiles: (**a**) different nanoparticles (**b**) various $\epsilon$ and $\phi$ (**c**) distinct M and Rd (**d**) different $\lambda$ and $\Delta$.

**Figure 3:**
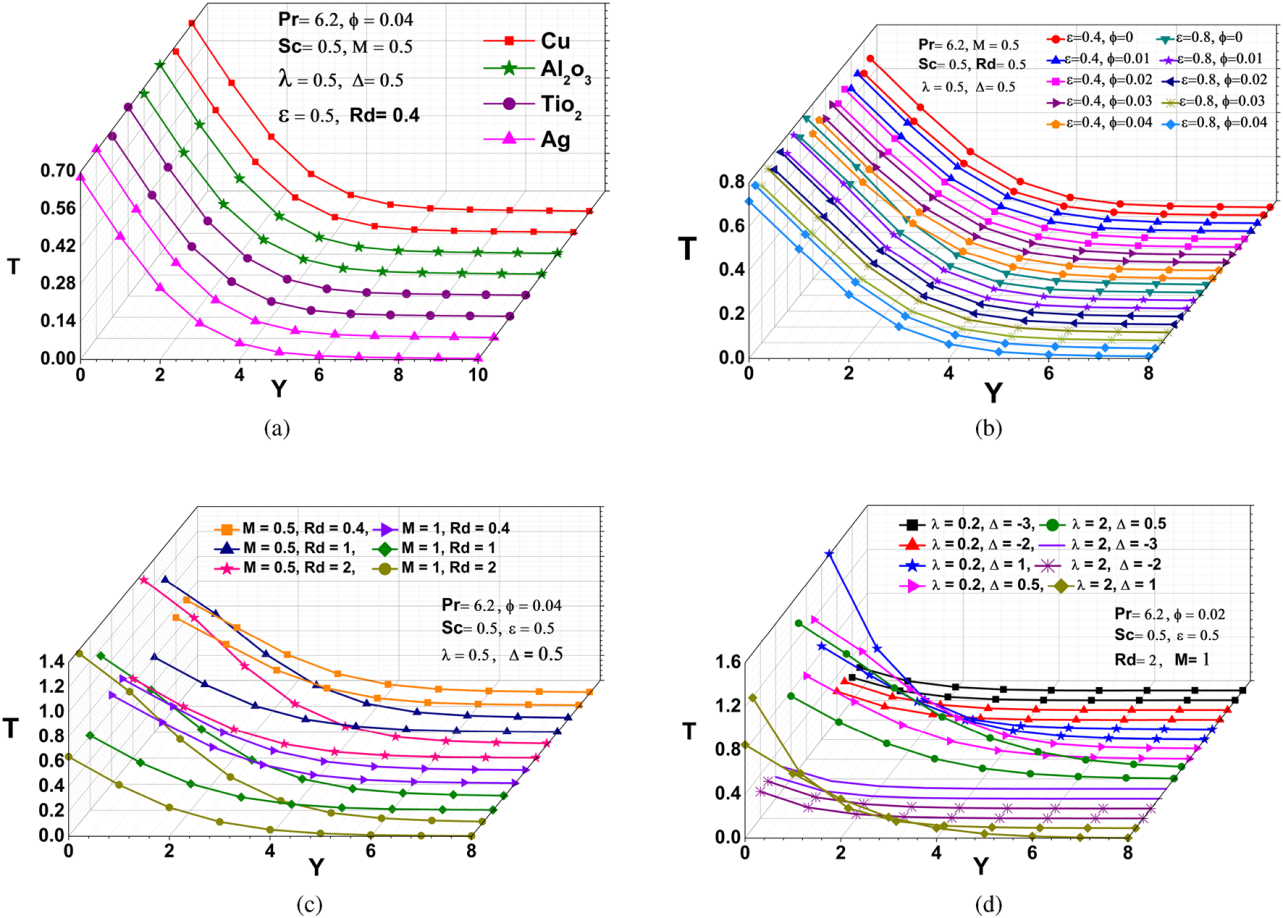
Temperature profiles: (**a**) different nanoparticles (**b**) various $\epsilon$ and $\phi$ (**c**) distinct M and Rd (**d**) different $\lambda$ and $\Delta$.

**Figure 4:**
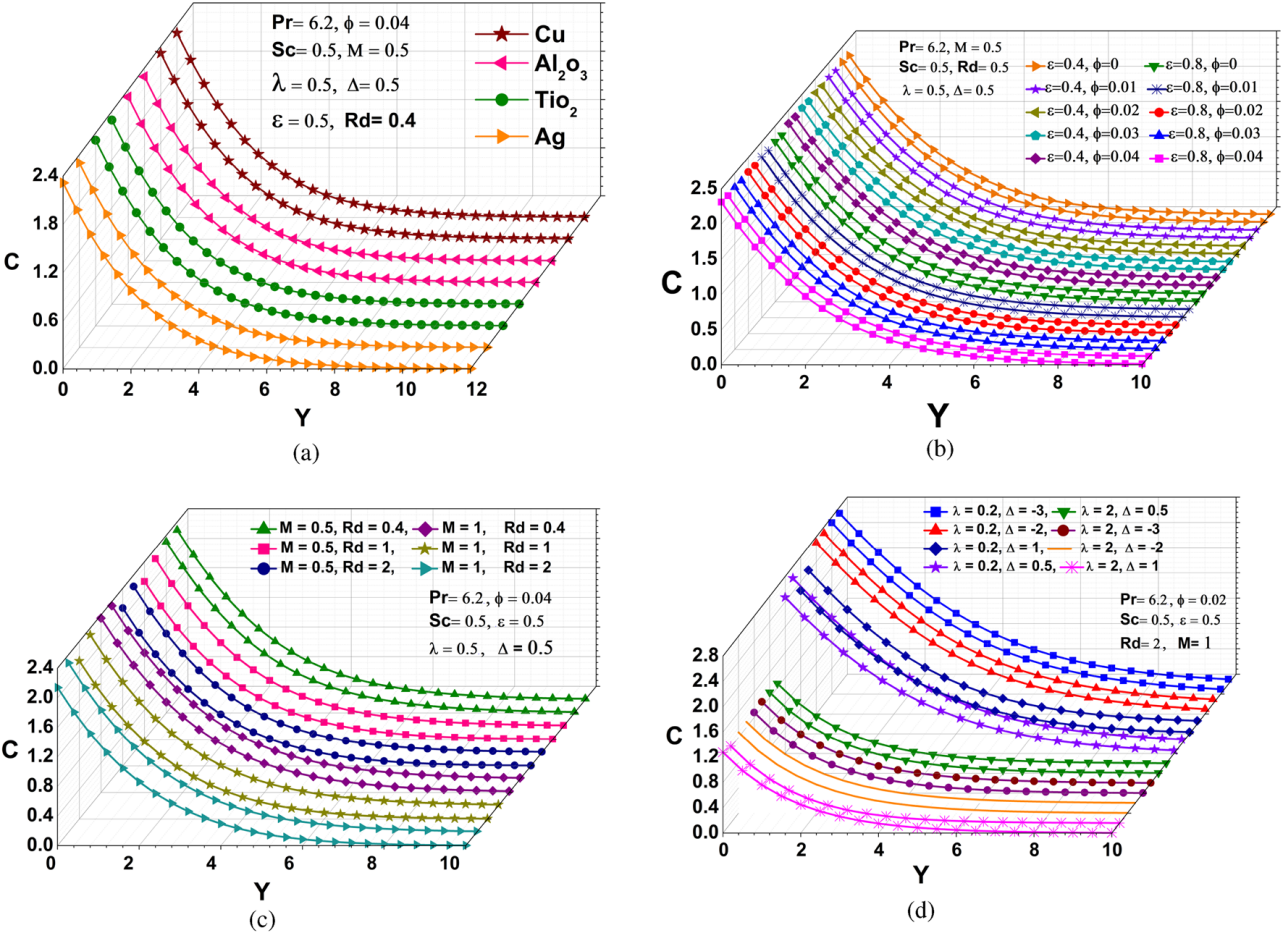
Concentration profiles: (**a**) different nanoparticles (**b**) various $\epsilon$ and $\phi$ (**c**) distinct M and Rd (**d**) different $\lambda$ and $\Delta$.

**Figure 5:**
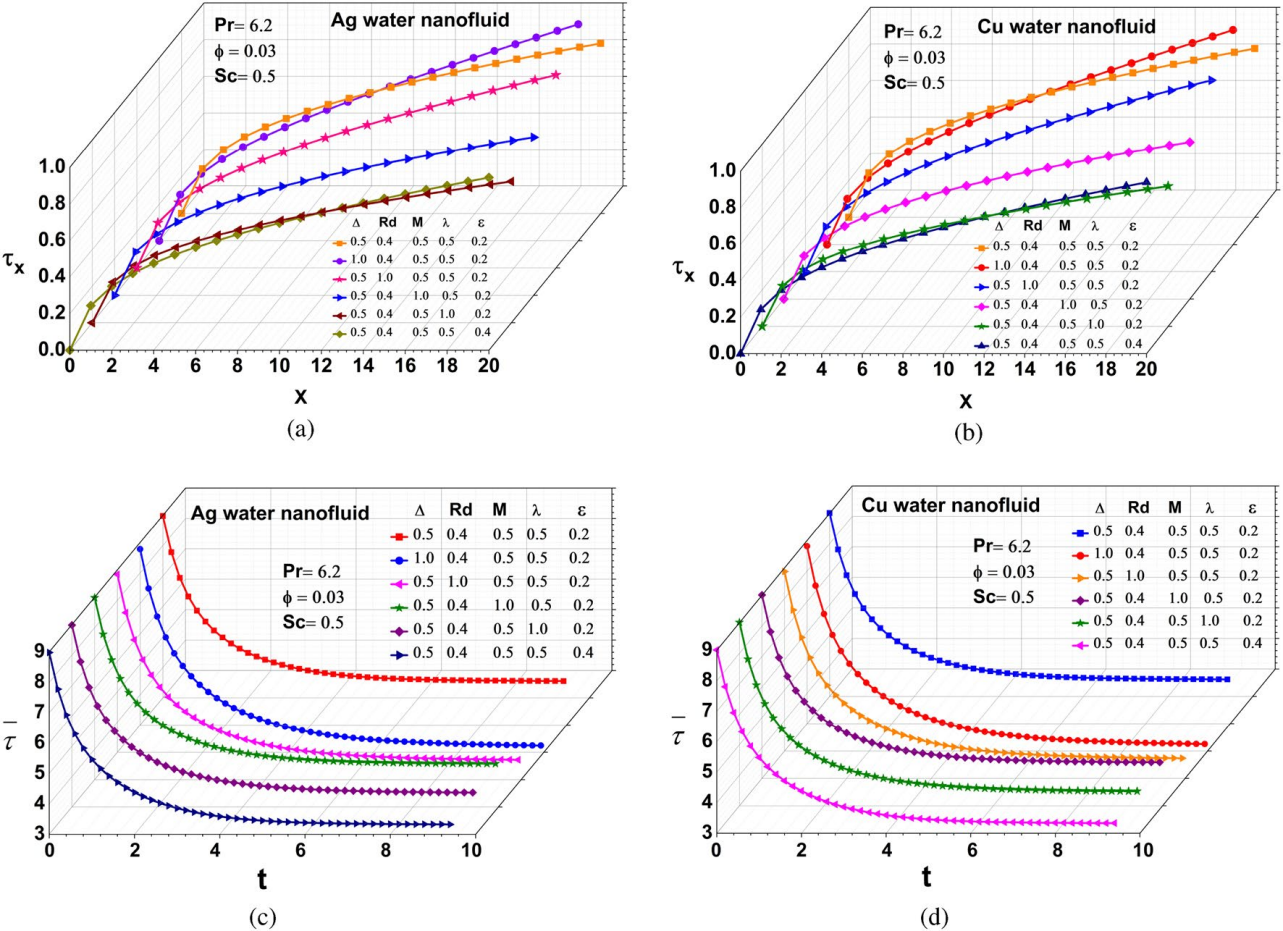
Local skin friction: (**a**) Silver (**b**) Copper, Average skin friction: (**c**) Silver (**d**) Copper.

**Figure 6:**
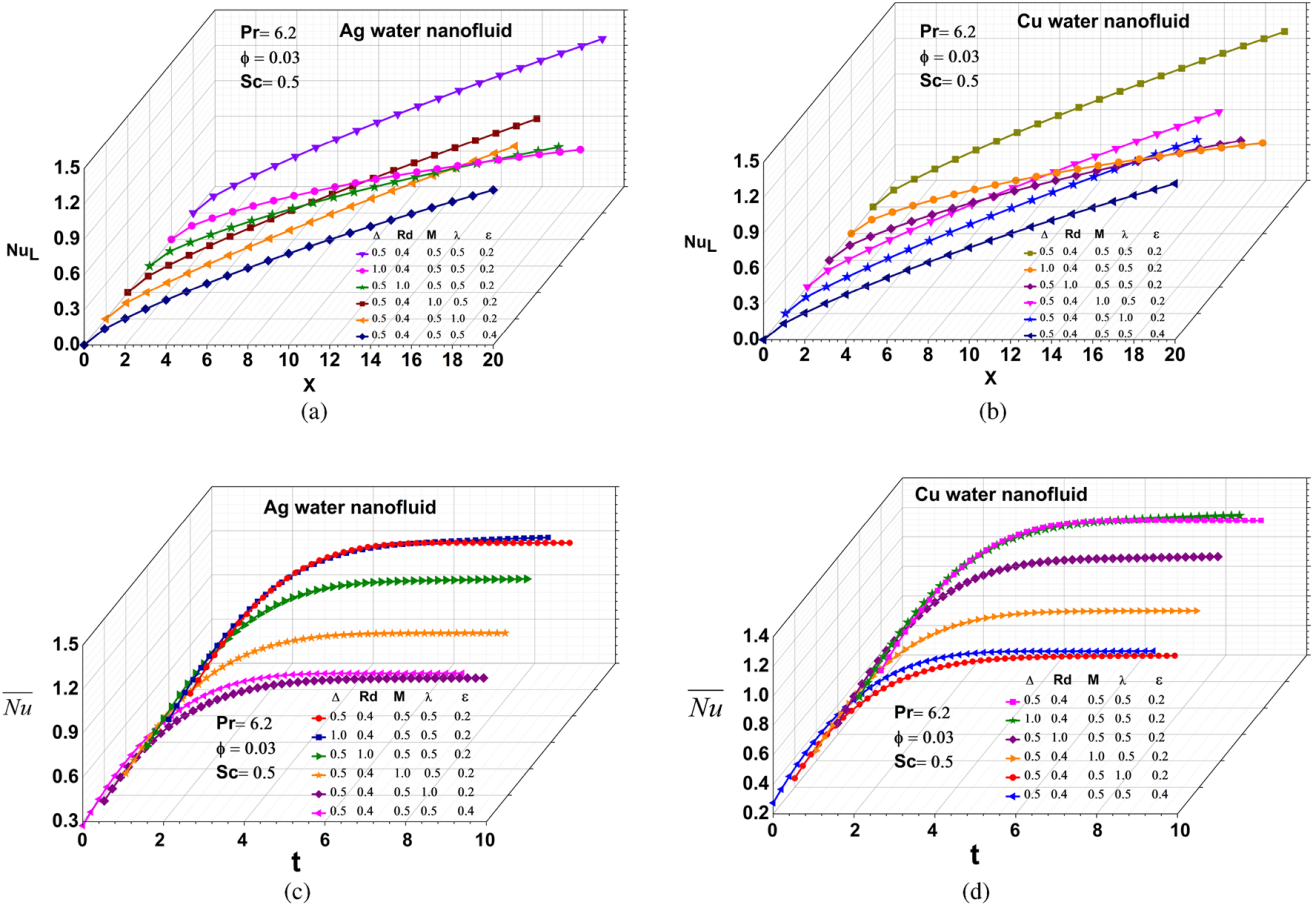
Local Nusselt number: (**a**) Silver (**b**) Copper , Average Nusselt number: (**c**) Silver (**d**) Copper.

**Figure 7:**
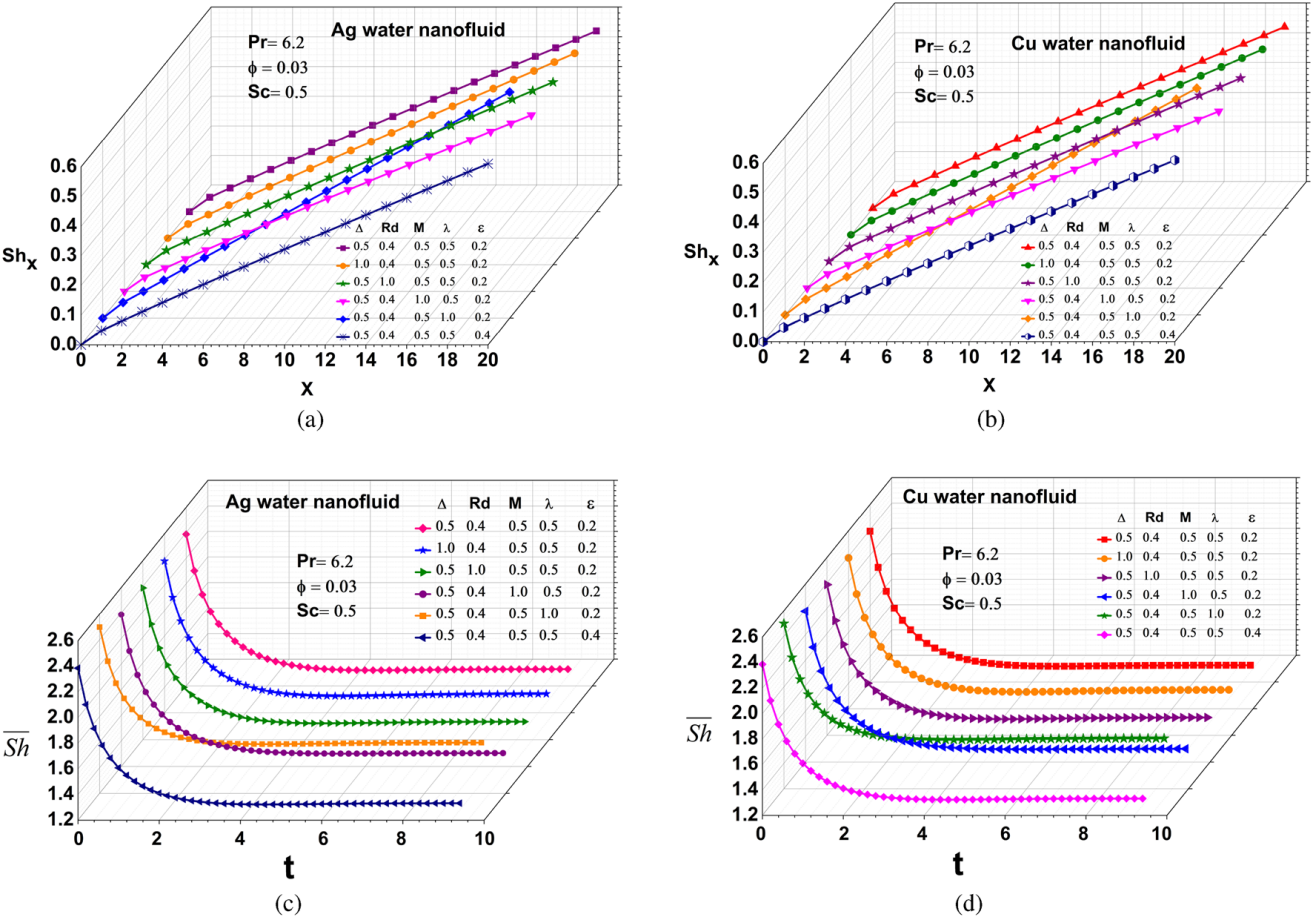
Local Sherwood number: (**a**) Silver (**b**) Copper, Average Sherwood number: (**c**) Silver (**d**) Copper.

In this work, four distinct kinds of nanoparticles Al$_{2}$O$_{3},$ Cu, Ag and TiO$_{2}$ as shown in Table 1 are tested by employing water as the base fluid. As shown in Figs. 2, 3, 4, 5, 6 and 7, spatial velocity, temperature and concentration inside the boundary layer are affected by the magnetic parameter (M), radiation parameter (Rd), heat source and sink ($\Delta$ ), nanoparticle volume fraction ($\phi$), viscous dissipation ($\epsilon$), chemical reaction ($\lambda$) and nanoparticle types. In Table 2, we present the temperature and the local skin-friction coefficient in response to changes in Prandtl number (Pr). The temperature and the local skin-friction coefficient consequently rise as Pr rises. Additionally, Table 3 demonstrates that when the Prandtl number grows, local skin friction and the local Nusselt number both rises.

**Table 2:** Temperature and local skin friction at steady state X = 1.0 compared with Lin^43^ at uniform surface heat flux, n = 0, M = 0.

Pr	Temperature		Local skin friction	
	Lin	Present result	Lin	Present result
0.72	1.7864	1.79450	1.2240	1.23555
1	1.6327	1.63984	1.0797	1.08992
2	1.3633	1.36909	0.8293	0.83726
4	1.1508	1.15581	0.6373	0.64343
6	1.0464	1.05082	0.5462	0.55138
8	0.9796	0.98352	0.4895	0.49399
10	0.9314	0.93494	0.4494	0.45351
100	0.5675	0.56512	0.1840	0.18437

**Table 3:** Local skin friction and Nusselt number at X = 1.0, steady state are compared with those of Hossain and Paul^44^ for different values of Pr, n = 0.5 and M = 0.

Pr	Local skin friction		Local Nusselt number	
	Hossain and Paul	Present result	Hossain and Paul	Present result
0.01	5.13457	5.15625	0.14633	0.14594
0.05	2.93993	2.95712	0.26212	0.26155
0.1	2.29051	2.30639	0.33174	0.33090

### Velocity distribution

Impact of radiation and MHD, different nanofluids, various volume fraction and dissipation as well as chemical reactions and heat source/sink on velocity profiles is seen in the Fig. 2(a–d). Using Fig. 2a, we can see that fluid velocity starts increasing with enhanced free convective heat transfer of different nanofluids due to their reduction potential. This will cause them to lose electrons to the new species. When silver (Ag) and copper (Cu) are compared, we can notice that copper(Cu) has a high velocity due to less viscous nature of copper water nanofluid. As the volume fraction of nanoparticles as well as viscous disipation increases, the velocity of fluid decreases and rises respectively due to the absorbent characteristics of the Composition of mixture containing nanoparticles as shown in Fig. 2b. Figure 2c displays the impacts of MHD and radiation over velocity field. Since the magnetic force is perpendicular to the momentum force and results in uniform circular motion, causes the reduction in momentum for the rise in MHD effect. In addition, the momentum wavelength and radiative force are directlty propositional to each other catalyzes the hike in momentum for higher radiative values. The behaviour of a chemical reaction and a heat source/sink is depicted in Fig. 2d. It is easy to observe how velocity decreases as $(\lambda )$ increases. It occurs because a larger $(\lambda )$ diminishes velocity of the reaction, slowing the particles and reducing the possibility of successful collisions between the reactant particles. Moreover, the heat source parameter ($\Delta$) effects on the velocity profiles. It is clear from Fig. 2d that, with an increase in the heat generation/absorption parameter, the boundary layer increases and as a consequence to increases the momentum boundary layer thickness. The velocity field increases as a result of a reservoir that supplies and absorbs energy in the form of heat for higher heat source/sink values.

### Temperature distribution

The effect of various nanofluids on dimensionless temperature is depicted in Fig. 3a. It has been discovered that the temperature decreases as the copper resistivity varies with temperature in a parabolic pattern. The graph clearly shows that when the resistance of copper increases, the temperature of copper rises somewhat more than that of other nanoparticles, reducing conductivity as the temperature rises. The influence of the nanoparticle volume fraction coefficient ($\phi$) and viscous dissipation ($\epsilon$) on the fluid temperature profile (T) is shown in Fig. 3b. It is noticeable that raising the nanoparticle volume fraction coefficient ($\phi$) as well as the increasing viscous dissipation parameter ($\epsilon$) rises the fluid temperature, because of the faster movement of nanoparticle in the fluids and the dissipation of some kinetic energy through viscosity. The impacts of MHD and radiation parameter at the thermal behavior is proven in Fig. 3c. It is able to be concluded from this figure that increasing rate in the wall temperature can be rises by incorporating MHD and thermal radiation effects because if a conductor is moved relative to a magnetic field, then a voltage is induced, which results in a flow of current between the terminals and the high-frequency electromagnetic radiation emitted by it is greater than the low-frequency radiation. The shorter wavelength occurs at higher frequencies. This means that the intensity of the radiation emitted is greater from a hotter body. The graph in Fig. 3(d) illustrates the consequences of the chemical reaction and heat source/sink parameter over the temperature profile. It is reported a significant increase in the amount of chemical reaction enhances the thickness of the thermal boundary layer since it raises the average kinetic energy of the reactant molecules. As a result, a higher fraction of molecules will possess the least amount of energy required for an effective collision and when the heat source/sink parameter is enhanced, a passive heat exchanger transfers the heat generated by a mechanical device to a fluid medium, where it is dissipated away from the device, permitting regulation of the device’s temperature, indicating that the temperature rises.

### Concentration profile

The concentration profile vs Y graph is described in Fig. 4(a-d), for different physical parameters (Pr, M, Rd, $\phi$, $\epsilon$ and Sc). A common result obtains from the assorted study of Fig. 4(a-d) that due to the nonuniform mass flux, the fluid has maximum concentration at a x = 1. Subfigures (a-d) of Fig. 4(a-d) are explained individually to demonstrate the effect of Pr, M, Sc, $\phi$, $\epsilon$ and Rd on the concentration profile, respectively. On the basis of Fig. 4a, it can be seen that the concentration profile near the cone has its minimum magnitude for silver (Ag) and maximum magnitude for copper (Cu), since the new species have a higher reduction potential causing electrons to be lost. The effects of the nanoparticle volume fraction parameter on the fluid concentration C for water-based nanofluids are shown in Fig. 4b. It is obvious that boosting the nanoparticle volume fraction parameter raises the fluid concentration. It is because the density of nanofluids increases as the volume fraction of nanoparticles increases, slowing down the nanofluid flow concentration and increasing the thickness of the species boundary layer, which enhances mass diffusivity and boosts the rate of surface heat transfer. The interaction of MHD and Rd on concentration profiles is presented in Fig. 4c. In these figures, it is obvious that as the values increase M and Rd, the species boundary peak thickness diminishes . The reason behind this is that involvement of a field of magnetism in an liquid that conducts electricity provides a force known as the Lorentz force, which opposes the flow of direction as well as causes depreciation in concentration profiles (Fig. 4c), while also requiring more energy from the fluid to counteract the drag force provided via Lorentzian retardation as well as the scattering by the atmosphere that results in the part of the total radiation that reaches the surface after changing directions. The impact of the chemical reaction as well as the heat source/sink parameters, on the concentration profile is depicted in Fig. 4d. Also the decline in concentration profile is followed by a rise in chemical parameter and fluctuation in concentration depends on increasing heat source/sink parameter can be seen in the Fig. 4d. On the other hand, the Sherwood number grows when the heat source/sink parameter is enhanced at $\lambda$ = 2 but diminishes when the source/sink parameter is elevated at $\lambda$ = 0.2, implying that the chemical reaction has a propensity to lower the mass transfer rate at the surface cone. The reason for this, when the concentration of all the reactants increases, more molecules or ions interact to form new compounds and the rate of reaction increases.

### Physical quantities of interest

On Figs. (5, 6 and 7) are plotted its effects on both local and average drag coefficient, local and average heat transfer rate and local and average mass transfer rate under various interesting parameters ($\Delta ,$ Rd, M, $\lambda ,$ and $\epsilon$).

#### Skin friction coefficient

The variation of local and average skin friction coefficient for Ag and Cu nanofluids for various values of $\Delta ,$ Rd, M, $\lambda ,$ and $\epsilon$ is illustrated in Fig. 5(a-d). The magnitude of the skin friction coefficient for Ag and Cu water-based nanofluids decays with varying parameters in this experiment, since the skin friction is caused when a fluid rubs against the surface of an element moving through it. It grows with the square of the velocity and is proportional to the area of the surface in contact with the fluid.

#### Heat transfer rate

The influence of various factors upon that local and average heat transfer rate of Ag and Cu liquid nanofluids is shown in Fig. 6(a-d). In this case, the local and average heat transfer rate against as for parameters change, but the reverse effect is observed with the supplied parameters because of the atomic chain behaviour, nanomaterials are properly diffused in the base fluid realised substantial benefits such as improved heat conduction, diminished possibilities of erosion and enhanced thermal conductivity and mixture stability.

#### Sherwood number

Figure 7(a-d) focus on the effect of changes in the values of $\Delta ,$ Rd, M, $\lambda ,$ and $\epsilon$ on the influence of local and average Sherwood numbers for Ag and Cu water-based nanofluids. The local and average Sherwood numbers decline as $\Delta ,$ Rd, M, $\lambda ,$ and $\epsilon$ increase.The parameters exhibit a reversal of the trend because the solute diffuses from a region of greater concentration to a region of lower concentration with a magnitude proportionate to the concentration gradient.

## Result findings

In terms of fluid velocity, temperature and concentration, it is obvious that copper provides superior heat and mass transmission in fluid motion than silver. This leads to an elevation in fluid velocity, temperature and concentration with respect to the viscous dissipation regardless of the volume fraction increase. As a result, we can infer that the momentum of the motion has a tendency to decrease at altitude, while the rate of thermal radiation has a tendency to rise. Similarly, when a chemical reaction happens at the velocity of a liquid, its value drops with regard to the heat source / sink and if the value of heat source / sink grows, the velocity rises with respect to the chemical reaction. It has been explained that the temperature rises when parameters such as MHD, Rd and heat source/sink values climb to the surface together whereas the heat flux rotates at x = 1. In terms of concentration, when the value of the parameters MHD, chemical reaction, thermal radiation and heat source/sink is enhanced, the concentration declines. Furthermore, depending on the magnitude of the chemical reaction, concentration fluctuations in x = 1 are abserved.

- It is reported that copper has a slightly greater heat transfer rate than silver for local as well as average Nusselt numbers.
- Silver is stated to have a little greater local wall shear stress than copper, whereas copper has a slightly higher wall shear stress than silver for average skin friction due to friction within the Cu and Ag.
- According to the study, copper generates a slightly greater mass transfer rate due to molecular diffusion than silver for both local and average Sherwood numbers.
- In future researchers can contribute towards the problem with Soret and Dufour effects.

## Data Availability

The datasets used and/or analysed during the current study available from the corresponding author on reasonable request.
